# Novel Wireless Bioimpedance Device for Segmental Lymphedema Analysis Post Dual-Site Free Vascularized Lymph Node Transfer: A Prospective Cohort Study

**DOI:** 10.3390/s21248187

**Published:** 2021-12-08

**Authors:** Chang-Cheng Chang, Wei-Ling Jan, Cheng-Huei Juan, Nai-Hsin Meng, Bor-Shyh Lin, Hung-Chi Chen

**Affiliations:** 1 Division of Plastic and Reconstructive Surgery, Department of Surgery, China Medical University Hospital, Taichung 404332, Taiwan; changcc1975@gmail.com (C.-C.C.); willyjan58@gmail.com (W.-L.J.); 2 School of Medicine, College of Medicine, China Medical University, Taichung 404333, Taiwan; 3 Institute of Imaging and Biomedical Photonics, National Yang Ming Chiao Tung University, Tainan 711010, Taiwan; 4 Institute of Biomedical Science, China Medical University, Taichung 404333, Taiwan; peishengskincare@gmail.com; 5 Department of Physical Medicine and Rehabilitation, China Medical University Hospital, Taichung 404332, Taiwan; d6351@mail.cmuh.org.tw; 6 International Medical Service Center, China Medical University Hospital, Taichung 404332, Taiwan

**Keywords:** bioimpedance, lymphedema, vascularized lymph node transfer

## Abstract

An innovative wireless device for bioimpedance analysis was developed for post-dual-site free vascularized lymph node transfer (VLNT) evaluation. Seven patients received dual-site free VLNT for unilateral upper or lower limb lymphedema. A total of 10 healthy college students were enrolled in the healthy control group. The device was applied to the affected and unaffected limbs to assess segmental alterations in bioimpedance. The affected proximal limb showed a significant increase in bioimpedance at postoperative sixth month (3.3 [2.8, 3.6], *p* = 0.001) with 10 kHz currents for better penetration, although the difference was not significant (3.3 [3.3, 3.8]) at 1 kHz. The bioimpedance of the affected distal limb significantly increased after dual-site free VLNT surgery, whether passing with the 1 kHz (1.6 [0.7, 3.4], *p* = 0.030, postoperative first month; 2.8 [1.0, 4.2], *p* = 0.027, postoperative third month; and 1.3 [1.3, 3.4], *p* = 0.009, postoperative sixth month) or 10 kHz current ((1.4 [0.5, 2.7], *p* = 0.049, postoperative first month; 3.2 [0.9, 6.3], *p* = 0.003, postoperative third month; and 3.6 [2.5, 4.1], *p* < 0.001, postoperative sixth month). Bioimpedance alterations on the affected distal limb were significantly correlated with follow-up time (*rho* = 0.456, *p* = 0.029 detected at 10 kHz). This bioimpedance wireless device could quantitatively monitor the interstitial fluid alterations, which is suitable for postoperative real-time surveillance.

## 1. Introduction

Lymphedema is caused by interstitial fluid accumulation due to obstruction of the lymphatic drainage system, resulting in swelling of the affected part. Clinical symptoms include pain, swelling, heaviness, skin atrophy, and recurrent cellulitis [1,2,3,4,5]. To relieve the swelling, conservative decongestive physiotherapies, such as wearing compression garments, exercise, and manual lymphatic drainage, are initially applied for lymphedema. However, various surgeries have been indicated for refractory lymphedema, including lymphaticovenular anastomosis (LVA), vascularized lymph node transfer (VLNT), suction-assisted lipectomy, radical reduction with preservation of perforators, and Charles’ procedure [1,2,3,4]. All therapeutic procedures intend to reduce limb volume, decrease episodes of infection, and improve the quality of life. Free VLNT is adopted when the above methods disclose ineffective outcomes. It is a novel approach, with the functional lymph nodes carried to an obstructed site, where the growth factors induce lymph angiogenesis and possible immunomodulation [1,3,4,5], hence, a change in fluid composition. Currently, we have had the clinical experience of using dual-site free VLNT and combined surgery with LVA or other excisional procedures to treat extremity lymphedema [1,2,6,7,8].

The common evaluation of lymphedema is to measure the limb volume change, for example, the time-consuming water displacement method and individual-dependent limb circumference reduction rate. The diagnosis of lymphedema is a relative 10% or 200 mL increase in volume, or 2 cm increase in circumference, compared with the unaffected limb or baseline of the affected limb [9,10,11]. Nevertheless, localized lymphedema may be misdiagnosed in the early stages since extracellular water only comprises 25% of the normal limb. Moreover, the two quantitative methods cannot represent the true limb volume and directly reflect the composition of the tissue. Changes in soft tissue components can act as camouflage, masking the volume change caused by lymphatic fluid accumulation, or the relief post therapeutic procedures [9,12,13]. Direct image assessment of dermal lymph flow and lymphatic channel patency by lymphoscintigraphy and indocyanine green (ICG) lymphangiography is valid and reliable for diagnosing lymphedema, ranging from subclinical or early to advanced stages. However, these approaches are only qualitative for post-treatment follow-up rather than quantitative assessment [14,15,16,17].

Bioimpedance analysis, which detects different conductivities of body tissues according to a specific current, can selectively measure and quantitatively reflect the accumulated fluid content of limbs. The accumulation of extracellular fluid, which conducts electricity more easily than fat and adipose tissue, decreases bioimpedance measurements [9,14,18]. Currently, there are three models of bioimpedance devices, including single-frequency bioelectrical impedance analysis, multiple-frequency bioelectrical impedance analysis, and bioimpedance spectroscopy (BIS). Bioimpedance consists of resistance (R), which results from total body water and reactance (X_C_) caused by the conduction delay of cell membranes. Both resistance and reactance are frequency dependent. The capacitance characteristics of cell membranes impede the current flow at extremely low frequencies and precipitate total conduction at infinitely high frequencies, which allows us to determine the ratio of extracellular water to intracellular water [19,20].

The current FDA-approved bioimpedance device (L-Dex U400, ImpediMed, Inc., Carlsbad, CA, USA) only reveals the whole limb bioimpedance measurement, and it is unable to represent the multiple preferred sites, for example, the different levels for circumference measurement, on the affected extremity. Since the proximal and distal limbs vary in lymphedema improvement after dual-site free VLNT treatment, the segmental assessment of lymphedema seems more favorable than whole limb assessment for postoperative follow-up. Brenda et al. determined that segmental BIS displayed an uneven pattern of lymphedema distribution, which enhanced the diagnosis of localized or early-stage lymphedema, and identified additional lymphedema patients compared with whole limb BIS. Moreover, segmental BIS provides a more accurate post-treatment evaluation than whole-limb BIS [21,22,23,24]. Nevertheless, the placement of several wired electrodes still limits the feasibility of segmental bioimpedance measurements, and it could be less practical for instant real-time detection.

Currently, there is no clinical gold standard to assess the measurements of the bioimpedance device on the sites of application. The current measurement modalities are incapable of precisely reflecting the severity of the disease or the effect of the surgery. With the advancement and diversification of surgical treatment methods, a more suitable and quantitative measurement tool for post-treatment disease tracking is needed. Thus, a segmental bioimpedance measurement device was developed for post-dual-site-free VLNT, which involves both proximal and distal flap inset sites. Herein, we applied this innovative wireless device to a cohort of patients who undergone dual-site free VLNT for lymphedema treatment. We aimed to develop a novel bioimpedance device that could segmentally assess the lymphedema condition for outcome surveillance after the operation and during rehabilitation programs.

## 2. Materials and Methods

This prospective cohort study was approved by the Institutional Review Board of the China Medical University Hospital (No. CMUH106-REC1-111). From October 2017 to October 2019, we randomly selected seven patients who were planning to receive dual-site free VLNT for the treatment of unilateral upper or lower extremity lymphedema at China Medical University Hospital. A total of 10 college students were also enrolled as normal healthy controls. Of the patients, three were diagnosed with primary lymphedema, and the other four were diagnosed with secondary lymphedema resulting from surgery for breast or gynecologic cancer. The gastroepiploic (five patients) or supraclavicular (two patients) vascularized lymph node flap was harvested and divided into two lymph node flaps, which were separately transferred to the proximal (cubital fossa/popliteal fossa) and distal (wrist/ankle) sections of the affected extremity [1] (Table 1). The exclusion criteria were juveniles (age < 20 years), inflammation or infection of the portion of the skin, lymphedema caused by systemic diseases, history of muscular dystrophies, extensive limb fibrosis (e.g., in burned patients), current use of diuretics, and previous operation for the edematous limb.

Using our wireless bioimpedance monitoring device, we measured the segmental bioimpedance of the affected limbs, and the contralateral unaffected limbs in the pre-operation period and at postoperative one, three, and six months. Segmental bioimpedances were measured at the midpoint of the medial side of the proximal limb (thigh/arm) and the distal limb (calf/forearm) with currents of different frequencies (1, 2, 3, 4, 5, 6, 7, 8, 9, 10, and 20 kHz) (Table 2). Normal bioimpedances correlated to different frequencies were obtained from 10 healthy college students without lymphedema as a control group. The circumferences of the proximal limb (thigh/arm) and distal limb (calf/forearm) were also recorded simultaneously (Table 2).

We sought to demonstrate the increasing bioimpedance and decreasing limb circumference at each follow-up point for the shifting of the accumulated extracellular fluid post dual-site-free VLNT. In addition, we counted the difference in bioimpedance between the affected limbs (proximal/distal) and the contralateral unaffected limbs (proximal/distal). Furthermore, we checked the bioimpedance of healthy limbs (proximal/distal) in the control group, which represented the maximum level that the measurements in patients would not attain.

### 2.1. Electrically Conductive Characteristics of Human Tissues

**Figure 1:**
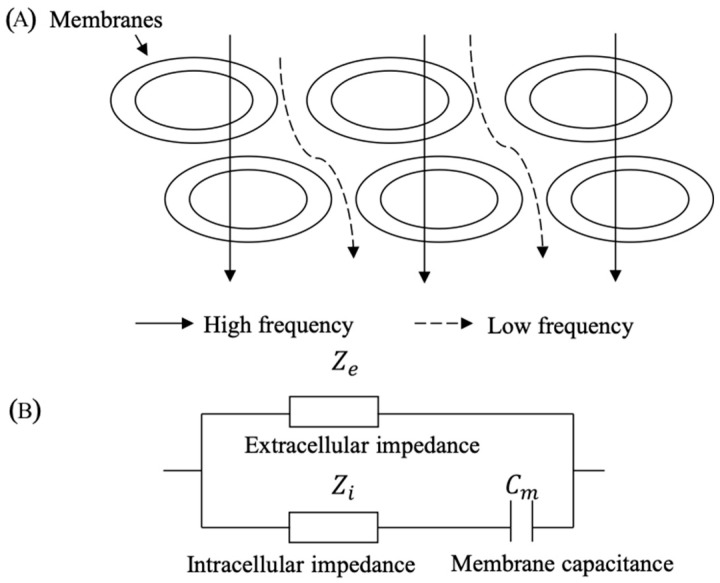
(**A**) Illustration for the pathway of electrical current with different frequencies in human tissues, and (**B**) electrically equivalent model of human tissues.

In human tissues, different tissue components contain different conductive properties. When an electrical current with a lower frequency passes through different human tissues, the conductive pathway of the electrical current is mainly along the extracellular space. Only higher-frequency electrical currents could pass through the cell membrane (Figure 1A). Generally, human tissues contain both resistance and capacitor properties simultaneously. Therefore, the bioimpedance of the human tissue changes with the variation in the electrical current frequency. The equivalent model of the human tissue contains the extracellular impedance ($Z_{e}$), intracellular impedance ($Z_{i}$), and membrane capacitance ($C_{m}$) (Figure 1B) [25,26].

### 2.2. Bioimpedance Monitoring Device

**Figure 2:**
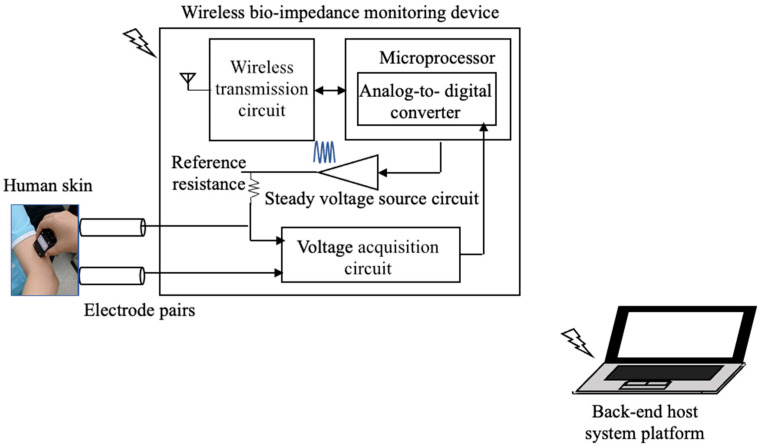
A wireless bio-impedance monitoring device consists of a pair of stainless-steel electrode probes and a wireless signal acquisition module. Our device measures approximately 6 cm × 3 cm.

The innovative wireless bioimpedance monitoring device is composed of a pair of stainless-steel electrode probes (distance between the electrodes: 1.0 cm) and a wireless signal acquisition module. The electrode probes were used to contact the human skin lightly to capture the bioimpedance information of the tissue. The wireless signal acquisition module is designed to produce a steady voltage current source (maximum voltage, 3 V; maximum current, 0.001 mA), with different frequencies, to extract the multi-frequency bioimpedance information and to transmit wirelessly to the back-end host system platform. The back-end host system platform receives the original bioimpedance information to calculate, display, and store the multi-frequency bioimpedances in real time. This device complies with the requirements of the Conformitè Europëenne (CE), and has received the CE safety certification; that is, it satisfies the basic requirements of product safety, proper protection of user health, and environmental protection. With the electrodes contacting the skin lightly at the thigh/arm and calf/forearm, the current frequencies from 1–20 kHz changed automatically within 1 s. The composition ratio of various tissues in the body can be measured realistically through the different conductivities of various tissues at different frequency currents [27] (Figure 2).

### 2.3. Statistical Analysis

The continuous data were presented as median and interquartile range due to the small sample size in the study. The bioimpedance and circumference data were analyzed using the generalized estimating equation (GEE), which included the intercept, main effect of time (as a categorical variable), main effect of limb type (affected vs. healthy), and two-way interactions of time by limb type. The change in bioimpedance and circumference from baseline to a later follow-up in each limb type was evaluated using the simple main effect of GEE. The difference in bioimpedance and circumference between limb types at each period was also assessed using the simple main effect of GEE. The relationship between time (as a continuous variable) and bioimpedance/circumference was assessed using the Spearman’s rank correlation. Finally, the consistency of the value change from baseline to a later follow-up between bioimpedance and circumference was evaluated using Spearman’s rank correlation. Statistical significance was set at *p* < 0.05 (two-sided), and no adjustment of multiple testing (multiplicity) was made in this study. Data analyses were conducted using SPSS version 22 (IBM SPSS Inc., Chicago, IL, USA).

## 3. Results

The demographic and clinical characteristics of the seven patients are presented in Table 1. The segmental tissue bioimpedance measurements, using frequencies of 1–10 kHz and 20 kHz, and the circumference measurement were investigated in the pre-operative period and in the postoperative first, third, and sixth months (Table 2).

**Figure 3:**
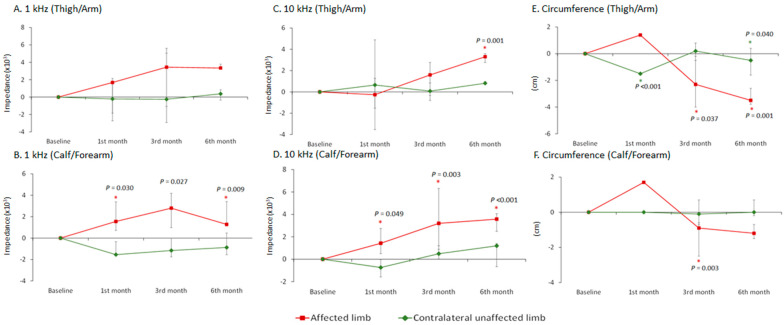
(**A**–**D**) Bioimpedance detection with 1 kHz and 10 kHz, compared with baseline, of the affected and contralateral unaffected limbs in the postoperative 1st, 3rd, and 6th months. A significant increase in bioimpedance of the proximal limb (thigh/arm) was observed via 10 kHz at six months after dual-site VLNT surgery. A significant increase in bioimpedance of the distal limb (calf/forearm) was observed via 1 kHz and 10 kHz after dual-site VLNT surgery. (**E**,**F**) Circumference measurement, compared with baseline, of the affected and contralateral unaffected limbs in the postoperative first, third, and sixth months. A significant decrease in circumference was observed after dual-site VLNT surgery. Data were presented as median, and the error bar denoted the interquartile range. The tests were made using the simple main effect of generalized estimating equation. * Statistical significance at *p* < 0.05.

**Figure 4:**
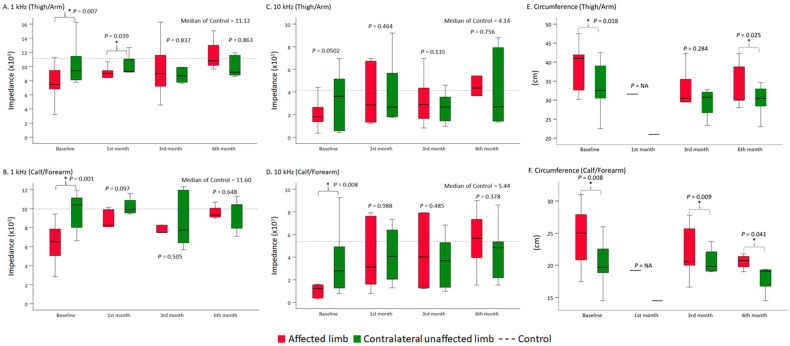
(**A**–**D**) Bioimpedance detection at 1 kHz and 10 kHz of the affected and contralateral unaffected limbs in the pre-operative period and postoperative first, third, and sixth months. There was no significant difference in the bioimpedance between the affected and contralateral unaffected limbs after dual-site VLNT surgery. The dotted line showed the median of healthy control group without lymphedema. (**E**,**F**) Circumference measurement of the affected and contralateral unaffected limbs in the pre-operative period and postoperative first, third, and sixth months. A significant difference in the circumference between the affected and contralateral unaffected limbs was still observed six months after dual-site VLNT surgery. Data were presented as median, and the error bar denoted the interquartile range. The tests were made using the simple main effect of generalized estimating equation. * Statistical significance at *p* < 0.05.

The affected proximal limb (thigh/arm) revealed an increased tendency of bioimpedance in the postoperative third (3.4 [−2.9, 5.6], *p* = 0.475) and sixth (3.3 [3.3, 3.8], *p* = 0.177) months at 1 kHz, although no statistical significance was observed (Figure 3A). The affected proximal limb (thigh/arm) showed a significant increase in bioimpedance during the postoperative sixth month (3.3 [2.8, 3.6], *p* = 0.001) at 10 kHz due to the better penetration of soft tissues (Figure 3C). Furthermore, the difference between the affected proximal limb (thigh/arm) and the contralateral unaffected proximal limb (thigh/arm) became insignificant after the dual-site free VLNT surgery, whether as detected at 1 kHz (postoperative third and sixth months) (Figure 4A), or at 10 kHz (postoperative first, third, and sixth months) (Figure 4C). The bioimpedance of the affected proximal limb (thigh/arm) was positively correlated with the postoperative follow-up time (*rho* = 0.272, *p* = 0.210 detected at 1 kHz; *rho* = 0.320, *p* = 0.136 detected at 10 kHz) (Table 3).

The bioimpedances of the affected distal limb (calf/forearm) significantly increased within six months after dual-site free VLNT surgery, whether passing with the 1 kHz currents (1.6 [0.7, 3.4], *p* = 0.030, postoperative first month; 2.8 [1.0, 4.2], *p* = 0.027, postoperative third month; and 1.3 [1.3, 3.4], *p* = 0.009, postoperative sixth month) (Figure 3B) or the 10 kHz current (1.4 [0.5, 2.7], *p* = 0.049, post-operative first month; 3.2 [0.9, 6.3], *p* = 0.003, postoperative third month; and 3.6 [2.5, 4.1], *p* < 0.001, postoperative sixth month) (Figure 3D). In addition, the difference between the affected distal limb (calf/forearm) and the contralateral unaffected distal limb (calf/forearm) became insignificant at post-operative first, third, and sixth months, whether as detected by 1 kHz (Figure 4B) or 10 kHz (Figure 4D). The alterations in bioimpedance of the affected distal limb (calf/forearm) were also positively correlated with the postoperative follow-up time (*rho* = 0.401, *p* = 0.058 detected at 1 kHz; *rho* = 0.456, *p* = 0.029 detected at 10 kHz). This indicates that the bioimpedance increases along with the decrease in interstitial fluid accumulation after dual-site free VLNT surgery (Table 3).

A significant improvement in the affected proximal limb (thigh/arm) (−2.3 [−4.0, −0.2], *p* = 0.037, postoperative third month; −3.5 [−3.8, −2.6], *p* = 0.001, postoperative sixth month) and the affected distal limb (calf/forearm) (−0.9 [−2.5, −0.6], *p* = 0.003, postoperative third month) after dual-site free VLNT surgery was observed via the circumference measurement (Figure 3E,F). The extent of improvement was presented as the circumference reduction rate (%), and both the affected proximal limb (thigh/arm) (−10.4 [−10.9, −6.3]%) and the affected distal limb (calf/forearm) (−3.9 [−7.3, −3.3]%) ameliorated in sixth months after the operation (Table 4). The circumference measurement gradually decreased within six months after dual-site free VLNT surgery (*rho* = −0.412, *p* = 0.090 of the affected proximal limb; *rho* = −0.169, *p* = 0.504 of the affected distal limb). When compared with the baseline, the decrease of the circumference measurement was inversely related to the increase of the bioimpedance in both the proximal (*rho* = 0.700, *p* = 0.188 detected at 1 kHz; *rho* = 0.200, *p* = 0.747 detected at 10 kHz) and distal limb (*rho* = 0.200, *p* = 0.747 detected at 1 kHz) (Table 3).

## 4. Discussion

The accuracy of bioimpedance remains contentious in the screening of lymphedema [12,14,28,29,30,31,32,33,34,35]. Previous studies demonstrated a low sensitivity (7.5–64%) of BIS in diagnosing breast cancer-related lymphedema [12,14,34,35], which was associated with 61–71% of positive predictive value and 67–70% of negative predictive value [35]. This may be caused by the easily manipulated localized swelling in early-stage lymphedema and the persistent proliferation of fibroadipose tissue in advanced-stage lymphedema [9,12,14,35]. Currently, data on the use of bioimpedance analysis in disease tracking after lymphedema treatment remains insufficient. Both Cho et al. and Cavezzi et al. considered that bioimpedance analysis is feasible for detecting slight extracellular fluid changes after complex decongestive therapy [36,37], and therefore, is practical for outcome monitoring. For surgical intervention of lymphedema, Sutherland et al. first utilized BIS to assess the effect of lymphovenous bypass for breast cancer-related lymphedema, and reported clinically significant improvement after lymphovenous bypass [38]. The above modalities measure the entire limb rather than the segmental measurement. However, the improvement of the extremities may be different at the proximal and distal ends. Hence, our research team previously proposed an innovative wireless bioimpedance monitoring device in 2018, during which it was applied to one female patient to demonstrate the upper limb lymphedema improvement at two weeks after receiving dual-site free VLNT [27].

Our innovative wireless bioimpedance monitoring device has several features. First, it can accurately reflect the degree of lymphedema at the preferred portion of the affected extremity for its single-point touch measurement and small electrodes that enable current signal circuiting within 1.0 cm. Second, with the portability of our wireless device, it is suitable as a take-home monitor for a rehabilitation program, which is crucial to lymphedema patients not only for conservative treatment, but also for postoperative follow-up. Third, our device is instant, easy to operate, and can show results in only 10 s [27], which is better than the long examination time of conventional imaging techniques such as lymphoscintigraphy and fluorescent lymphangiography. Therefore, it is not restricted by the venue or operator.

**Figure 5:**
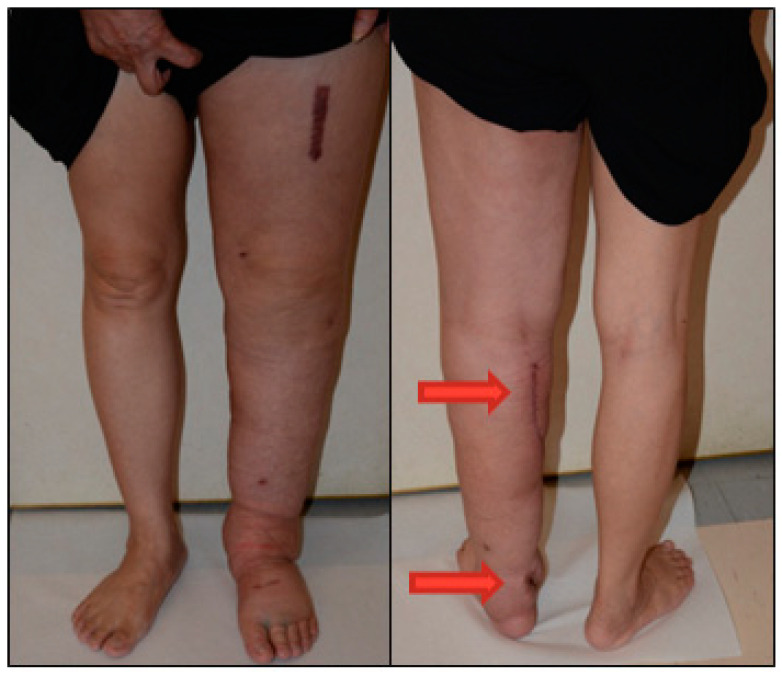
A patient suffered from lymphedema of the left lower limb. The dual-site free VLNT surgery was performed with two gastroepiploic lymph node flaps transferred to the popliteal fossa and ankle respectively (red arrow). The swelling of the left lower limb improved six months after surgery.

We confirmed that dual-site lymph node transplantation could effectively improve the lymphedematous condition probably caused by the interstitial fluid elimination. Thus, our hospital currently uses gastroepiploic lymph node flaps for selective lymphedema patients, which not only provides a large number of lymph nodes suitable for dual or multiple transplants, but also avoids iatrogenic donor site lymphedema (Figure 5). The dual-site (proximal plus distal) transplantation had synergetic effects in our cohort follow-up for better improvement of distal limb lymphatic drains than proximal ones. The bioimpedance detected at 10 kHz in the proximal limb significantly increased within six months postoperatively. A frequency of 10 kHz has better penetration of soft tissues, making it befit of the detection at the relatively thicker proximal part (arm/thigh). Although a frequency of 1 kHz has less tissue penetration, it is more sensitive in terms of reflecting the bioimpedance ratio [21,22]. Hence, multiple current frequencies could assist investigators in determining the whole picture of the fluid distribution on the limb.

The unaffected limb of the lymphedema patients may have the disease with only mild or no symptoms, and the impedance may fluctuate with posture, lifestyle, or rehabilitation program. Hence, there are fluctuations in the bioimpedance of the contralateral unaffected limb in our study. Nevertheless, most of them displayed no significant changes (Figure 3). The increase in bioimpedance should be an indicator of lymphedema reduction. However, there was a lack of significant correlation between reduction in limb circumference and increase in limb bioimpedance in our study. Since the circumference measurement cannot reflect the practical ratio of extracellular water to intracellular water, the result could be interfered easily with the changes in soft tissue components, causing it difficult to evaluate localized lymphedema and demonstrate the improvement and severity of lymphedema.

This study has several limitations. First, the number of dual-site VLNT surgery is still short, so we only investigate the difference through the proximal limb (thigh/arm) and distal limb (calf/forearm) in this study. Perhaps we can consider subdividing it into four groups (thigh, arm, calf, and forearm) in the future. In addition, the very small sample size may affect the reliability of the study. Second, our bioimpedance device detects only segmental bioimpedance to represent the water volume distribution. The device cannot analyze the nutritional status of the patient and the soft tissue composition, including adipose and muscle tissues. Third, the detection depth of our device would depend on the adjustment of the frequency; thus, the tomography imaging method could be developed with multi-segment measurement in future studies. We may examine multi-segment measurements to display the actual water distribution pattern of the entire extremity in the future. Fourth, a correlation between the bioimpedance method and image assessment, such as lymphoscintigraphy, ICG lymphangiography, and magnetic resonance lymphangiography, is still lacking. Fifth, we recommend using our device for postoperative surveillance and rehabilitation tracking. Patients with severe fibrosis may not be suitable due to the lack of deeper penetration. Sixth, there is no precision value (mL) for volume corresponding to the bioimpedance at present. Seventh, the performance metrics of our bioimpedance device in terms of accuracy and precision of measurement against known impedances should be further investigated. The application of this innovative bioimpedance device for long-term monitoring of lymphedema treatment outcomes still needs to be validated.

## 5. Conclusions

The dual-site free VLNT treatment is a promising method to improve refractory limb lymphedema by enhancing the lymphatic drainage. Our innovative wireless bioimpedance monitoring device demonstrated clinically significant and continuous improvement in lymphedema after dual-site-free VLNT treatment. The increase in bioimpedance after dual-site free VLNT treatment correlated with a decrease in circumference. The novel wireless bioimpedance device was proven to segmentally assess the lymphedema condition quantitatively by truly reflecting the fluid volume distribution in the limbs. It could also be an optimal evaluation for outcome surveillance during rehabilitation programs.

## Figures and Tables

**Table 1:** Demographic and clinical characteristics of the patients (*N* = 7).

No.	Sex	Age	BMI	Etiology	Symptom Duration (Months)	Affected Limb	Dual-Site VLNT	Hospital Stay (Days)	Follow-Up (Weeks)
1	F	66	27.43	Cervical cancer	84	LLL	Gastroepiploic	8	27
2	M	48	29.4	Unknown	24	RLL	Gastroepiploic	13	7
3	F	67	39.74	Unknown	12	LLL	Supraclavicular	15	8
4	F	42	31.16	Unknown	240	LLL	Gastroepiploic	16	27
5	F	72	44.43	Breast cancer	3	LUL	Gastroepiploic	34	30
6	F	63	28.25	Breast cancer	36	LUL	Gastroepiploic	15	25
7	F	51	24.17	Endometrial cancer	2	LLL	Supraclavicular	14	27
Avg.		58.43	32.08		57.29			16.43	21.57
SD		11.33	7.28		85.36			8.18	9.73

BMI: body mass index; VLNT: vascularized lymph nodes transfer; M: male; F: female; Avg.: average; SD: standard deviation; LLL: left lower limb; RLL: right lower limb; LUL: left upper limb.

**Table 2:** Detailed data of the bioimpedance under frequencies of 1 kHz and 10 kHz, and the circumference (*N* = 7).

	Baseline		1st Month		3rd Month		6th Month	
	AL	UL	AL	UL	AL	UL	AL	UL
Bioimpedance of 1 kHz PL	7.5	9.4	9.0	9.2	9.0	8.7	10.7	9.2
	[6.3, 11.3]	[7.8, 11.9]	[8.4, 9.4]	[9.2, 9.4]	[7.2, 11.6]	[7.8, 9.9]	[9.7, 11.0]	[8.8, 11.6]
Bioimpedance of 1 kHz DL	6.5	10.4	8.3	9.7	7.9	7.8	9.2	10.4
	[4.1, 7.9]	[7.8, 11.3]	[8.1, 9.9]	[9.4, 10.1]	[7.5, 8.3]	[6.4, 11.9]	[9.0, 9.4]	[7.9, 10.4]
Bioimpedance of 10 kHz PL	1.8	3.6	2.9	2.7	2.9	2.7	4.4	2.7
	[1.1, 3.5]	[0.5, 5.3]	[1.3, 6.7]	[1.8, 5.7]	[1.7, 4.4]	[1.4, 3.6]	[3.7, 5.4]	[1.4, 7.9]
Bioimpedance of 10 kHz DL	1.3	2.8	3.1	4.1	4.0	3.7	5.7	4.8
	[0.4, 1.6]	[1.2, 5.7]	[1.6, 7.6]	[2.0, 6.4]	[1.3, 7.9]	[1.3, 5.3]	[4.0, 7.3]	[2.2, 5.4]
Circumference of PL	41.0	32.6	31.6	21.0	30.5	31.5	30.0	30.5
	[31.8, 42.5]	[28.9, 41.6]	[31.6, 31.6]	[21.0, 21.0]	[29.5, 35.5]	[30.0, 32.8]	[30.0, 38.8]	[28.4, 33.0]
Circumference of DL	25.0	19.7	19.2	14.5	20.6	19.2	21.0	19.2
	[20.5, 30.3]	[18.5, 24.4]	[19.2, 19.2]	[14.5, 14.5]	[20.0, 25.7]	[19.0, 20.5]	[20.5, 21.8]	[19.0, 19.4]

AL: affected limb; UL: unaffected limb; PL: proximal limb (thigh/arm); DL: distal limb (calf/forearm). Unit of bioimpedance: Ω; Unit of circumference: cm Data were presented as median (25th percentile, 75th percentile).

**Table 3:** Correlation of measure rate derived from different measurements.

	*rho ^#^*	*p*
Affected proximal limb (thigh/arm)		
1 kHz Bioimpedance vs. Time	0.272	0.210
10 kHz Bioimpedance vs. Time	0.320	0.136
Circumference vs. Time	−0.412	0.090
1 kHz Bioimpedance difference vs. Circumference difference	0.700	0.188
10 kHz Bioimpedance difference vs. Circumference difference	0.200	0.747
Affected distal limb (calf/forearm)		
1 kHz Bioimpedance vs. Time	0.401	0.058
10 kHz Bioimpedance vs. Time	0.456	0.029 *
Circumference vs. Time	−0.169	0.504
1 kHz Bioimpedance difference vs. Circumference difference	0.200	0.747
10 kHz Bioimpedance difference vs. Circumference difference	0.000	1.000

^#^ Spearman’s correlation coefficient. * Statistical significance at *p* < 0.05.

**Table 4:** Circumference reduction rate (*N* = 7).

	Reduction Rate (%)
Affected limb	
Thigh/Arm	−10.4 [−10.9, −6.3]
Calf/Forearm	−3.9 [−7.3, −3.3]
Contralateral unaffected limb	
Thigh/Arm	−1.7 [−5.0, 1.2]
Calf/Forearm	0.0 [−1.0, 3.4]

Data were presented as median (25th percentile, 75th percentile). Circumference reduction rate (%) from baseline to sixth month = [(postoperative limb—preoperative limb)/preoperative limb] × 100.

## Data Availability

The data presented in this study are available upon request from the corresponding author.
